# Reevaluating the Importance of Informed Consent in Japan

**DOI:** 10.31662/jmaj.2024-0167

**Published:** 2025-08-01

**Authors:** Tatsuma Serge Yanagimoto, Rintaro Imafuku, Takuya Saiki, Mentor Ahmeti

**Affiliations:** 1 General Surgery, University of North Dakota School of Medicine and Health Sciences, Grand Forks, North Dakota, USA; 2 Medical Education Development Center, Gifu University, Gifu, Japan; 3 Trauma Acute Care Surgery, General Surgery, Sanford Health, Fargo, North Dakota, USA

**Keywords:** informed consent, patient autonomy, foreign patient, cultural nuances, Japanese medical ethics

## Abstract

Informed consent, a recent focus in the Japanese medical field, has been gaining ever-increasing attention and significance amid growing globalization. However, its implementation has introduced unique challenges stemming from cultural nuances, and differences in interpretations. The translation of “informed consent” as “explanation and agreement/consent” has created linguistic and conceptual hurdles among physicians, impeding effective patient-provider communication. As Japan’s medical ethics landscape progressively aligns with American and European standards, there is an urgent need for a thorough reconsideration of the meaning and significance of informed consent. This is further accentuated by the rising influx of foreign medical patients, emphasizing the necessity for a comprehensive reassessment of its true meaning and application. To address these challenges, a two-pronged approach could be highly effective. First, integrating the definition, evolution, and practical application of informed consent into medical school curricula would provide future physicians with a solid ethical foundation. Second, establishing a periodic informed consent training program, similar to certifications such as Basic Life Support would ensure that practicing physicians stay up-to-date with evolving global standards and best practices.

Japan’s medical ethics have undergone significant transformation in recent decades and are on track for further evolution, particularly in light of the substantial increase in foreign patients post-COVID-19 ^(1)^. Despite this progressive shift, the concept of informed consent within the medical field appears to have lagged amidst these changing ethical paradigms. Based on the author’s experiences, this discrepancy is particularly evident in scenarios involving the U.S. military population stationed in Japan, who frequently require medical care in Japanese hospitals. These patients often expect detailed explanations of various treatment options and an interactive dialogue with physicians to fully understand their situation. However, this process is often abbreviated, partly due to language barriers and differing interpretations of the concept of informed consent. The informed consent process presents numerous challenges, making the relationship between local hospitals and U.S. military medical care providers less fluid.

Historically, the principle of informed consent finds its roots in the early 20^th^ century, shaped by four pivotal judicial decisions―*Mohr v. Williams* (1905), *Pratt v. Davis* (1906), *Rolater v. Strain* (1910), and *Schloendroff v. Society of New York Hospital* (1914)―which established the foundation for patient autonomy. This evolution was further influenced by major international milestones in research ethics, including the Nuremberg Code (1947), the Declaration of Helsinki (1964, with subsequent revisions), and the Belmont Report (1979).

In Japan, however, the idea of informed consent emerged in the 1980s following a series of 1970s court cases highlighting the challenges arising from insufficient pre-operative communication between patients and physicians. In 1990, “setsumei to doi” (explanation and agreement/consent) officially became the Japanese translation for informed consent, aiming to seamlessly integrate the concept into the country’s medical practices while maintaining harmony with its cultural norms. Nevertheless, this decision, though potentially justifiable at the time, has since led to confusion and contemporary challenges amidst the evolving landscape of Japan’s medical ethics.

## An Inadequate Translation

Modern Japanese medicine grapples with a significant challenge stemming from the inadequate translation of “informed consent” into “setsumei to doi.” This issue is rooted in the insufficient education of physicians on the true meaning and scope of “informed consent,” as well as a lack of awareness that the concept was imported, modified, and translated into “explanation and agreement/consent. ” This misunderstanding is further compounded by Japan’s historical reliance on a paternalistic medical approach, where interactions between physicians and patients were predominantly unilateral, with physicians making decisions and providing information in a one-way manner ^(2)^.

In this context, the linguistic and conceptual confusion between “informed consent” and “setsumei to doi” becomes particularly problematic. While “informed consent” involves a dynamic, reciprocal process―including six critical steps: determination of competency and decision-making capacity, confirmation of voluntariness, disclosure, recommendation, and decision ^(3)^ ―“setsumei to doi” places disproportionate emphasis on the obligation of explanation. This translation fails to encapsulate the full breadth of informed consent, particularly the essential interactive exchange and collaborative dialogue between the physician and the patient.

By perpetuating a model that prioritizes explanation over mutual understanding, this misinterpretation undermines efforts to transition from the historically paternalistic approach to a more patient-centered model. Consequently, the development of meaningful interactions that respect patient autonomy and foster shared decision-making remains hindered, reflecting broader challenges in adapting Japan’s medical ethics to contemporary standards.

## A Gradual Shift toward Westernization of Medical Ethics

The evolving trend toward westernization in Japan’s medical ethics exacerbates the challenges stemming from the aforementioned misunderstanding. As the country gradually aligns itself with Western medical practices, the significance of clear communication and informed consent becomes even more pronounced. Reflecting on the 1990s, when less than 30% ^(4)^ of cancer patients in Japan were informed of their diagnosis, it indicated a limited proactive involvement of patients in their treatment decisions. However, this scenario has dramatically changed, with the disclosure rate reaching 94% in 2016 ^(5)^, showcasing a remarkable surge in patient engagement. In 2017, the Japan Medical Association General Policy Research Organization conducted the “Japanese Medical Awareness Survey” with 4,000 randomly selected Japanese adults, focusing on decision-making during serious illnesses. Nearly 75% of respondents leaned toward a shared decision-making model, indicating an awareness of the crucial role of self-determination in healthcare ^(6)^.

This transformation is also influenced by Japan’s evolving social structure, which has contributed to a broader transition from family-centered decision-making, rooted in collectivist traditions, to an approach that prioritizes individual autonomy in medical choices ^(7)^. These developments illustrate not only a move away from paternalism toward a collaborative, shared decision-making model but also a deeper cultural shift where patients are increasingly recognized as independent agents in their healthcare decisions.

The move toward patient-centered care necessitates a heightened emphasis on fostering open communication and active involvement in medical decision-making processes. All these transformations suggest that the traditional translation of “informed consent” as “explanation and agreement/consent” may now be outdated, signaling the need for a reevaluation of what “informed consent” means for the Japanese population.

## An Upcoming Challenge: Accommodating Foreign Medical Patients

Despite the steady increase in Japan’s foreign population and the gradual return of short-term visitors post-COVID-19, the Japanese medical system remains largely unprepared to handle the treatment of foreign patients. A 2022 survey from the Japanese Ministry of Health, Labour and Welfare indicated that 91.5% of hospitals lacked understanding regarding the data on health care visits and associated policies for foreign patients. The survey also hinted toward the fact that a large number of hospitals were still ill-equipped regarding linguistic support ^(8)^. This lack of preparedness becomes especially problematic in the context of informed consent. Foreign patients, often hailing from diverse cultural backgrounds, bring different expectations regarding medical treatment. Within this unprepared healthcare landscape, the issues surrounding informed consent, including the nuanced concept of “explanation and agreement/consent” specific to Japan, are exacerbated. The potential for misunderstandings and complications is heightened, emphasizing the urgent need for improved cultural competence and communication strategies within the Japanese medical system to ensure effective and ethical treatment of all patients, regardless of their background.

Under these circumstances, it is crucial to develop a medical school curriculum that addresses the evolving demographics of Japan’s patient population and their unique tendencies. Ideally, a curriculum that teaches informed consent on a yearly basis, combined with patient simulation sessions in the latter years of medical school, would allow students to engage in practical scenarios with patients from different cultural backgrounds. This approach would better prepare medical students to navigate the complexities of informed consent and patient communication, ensuring they are more equipped when they graduate. The curriculum should also include a thorough exploration of the history of informed consent in Japan, its current interpretation, and its practical application in clinical settings. These educational enhancements would equip future physicians with a deeper understanding of Japan’s shifting medical landscape and emphasize the critical importance of nuanced, patient-centered communication and meaningful engagement.

As globalization brings a growing influx of ideas and individuals from diverse cultures, the landscape of medical ethics―particularly in the domain of informed consent and shared decision-making―continues to evolve rapidly. In this context, it is essential for the Japanese medical system and its physicians to understand the historical origins of informed consent and its current definition, ensuring that the process is effectively tailored to meet the needs of today’s diverse patient population.

One potential solution is to phase out the use of the term *setsumei to doi* in favor of adopting the English term “informed consent,” which more accurately reflects the concept’s comprehensive and interactive nature. Additionally, introducing a mandatory informed consent training program, similar to Basic Life Support or Advanced Cardiovascular Life Support courses, could prove invaluable. Such a program, beginning in medical school and requiring periodic renewal, would ensure that physicians remain well-versed in the latest developments and best practices, ultimately fostering a more patient-centered approach to care.

## Article Information

### Conflicts of Interest

None

### Author Contributions

*All Co-authors have met all four of the below ICJME criteria:

- Substantial contributions to the conception of the work

- Critical review of the work

- Final approval of the version

Agreement to be accountable for all aspects of the work in ensuring that questions related to the accuracy or integrity of any part of the work are appropriately investigated and resolved.

### Approval by Institutional Review Board (IRB)

Exempt from IRB approval

### Disclaimer

Rintaro Imafuku is one of the Editors of JMA Journal and on the journal’s Editorial Staff. He was not involved in the editorial evaluation or decision to accept this article for publication at all.
